# Neuropsychiatric events in an adult patient with influenza a (H3N2) treated with oseltamivir (Tamiflu): a case report

**DOI:** 10.1186/s12879-019-3827-4

**Published:** 2019-03-04

**Authors:** Ruochan Chen, Zhixiong Fang, Yan Huang

**Affiliations:** 1 Department of Infectious Disease, Xiangya Hospital, Central South University, 87 Xiangya Road, Changsha, Post Code 410008 Hunan China; 2 Hunan Key Laboratory of Viral Hepatitis, Department of Infectious Disease, Xiangya Hospital, Central South University, 87 Xiangya Road, Changsha, Post Code 410008 Hunan China; 3 Department of Infectious Disease, Xiangtan Central Hospital, 120 Heping Road, Xiangtan, Post Code 411100 Hunan China

**Keywords:** Oseltamivir, Neuropsychiatric events, Side effects, Adult

## Abstract

**Background:**

Total four influenza pandemics have happened since twentieth century, and currently influenza is in the seasonal epidemic around the world, with a sharp increase in its incidence each year. As an effective treatment of influenza infection, the use of oseltamivir is climbing globally. However, there have been reports of neuropsychiatric events associated with the drug, mainly in young patients and males. To our knowledge, this is the first reported case of oseltamivir-associated neuropsychiatric events occurring in an patient over 50-year-old.

**Case presentation:**

Here we present the case of a 57-year-old Chinese female with H3N2 influenza infection who developed abnormal psychiatric symptoms after administration of high doses of oseltamivir. The patient recovered completely within the cessation of oseltamivir.

**Conclusions:**

We hope that our case report will lead clinicians to be mindful about oseltamivir’s potential neuropsychiatric side effects, and to pay special attention to each patient’s mental state, both in children and adults.

## Background

Seasonal influenza is an acute respiratory infection caused by the influenza virus, generally arising annually in winter and spring each year. Currently the world’s only oral neuraminidase inhibitor drug, oseltamivir has long been recognized as one of the most effective influenza drugs in the world. In recent years, there have been several case reports of neuropsychiatric events occurring during, or after oseltamivir treatment in Japan and other countries. These side effects have been reported in children, adolescents and young adults in particular [1–4], and more frequently in males than females. However, few cases about the neuropsychiatric side effects of oseltamivir have been reported in adults. Here we present the case of a 57-year-old Chinese female with H3N2 influenza infection who developed abnormal psychiatric symptoms after administration of high doses of oseltamivir.

## Case presentation

A 57-years-old female was admitted to the department of infectious diseases in our hospital with symptoms of cough and expectoration, aggravating with fever and dyspnea for more than 10 days. This patient was retired and she kept chickens at home; she had a history of thyroma and hypertension for an unknown period of time. She denied any history of prior lung disease or neurological illness.

She had complained of cough with white sputum after catching a cold 10 days before admission to the hospital, with no chest discomfort or dyspnea. Her symptoms became worse with the onset of a high fever (up to 39 degrees Celsius), accompanied by aches and pains all over the body and loss of appetite. She underwent treatment with cefotaxime sodium (2.0 g, q12H) for 3 days in a local clinic, however her symptoms continued to worsen and she was transferred to a general hospital. There she tested positive for influenza A virus infection on a rapid diagnostic test. The doctor suspected a severe influenza A virus infection and prescribed 150 mg of oseltamivir to be taken orally twice daily. She concomitantly received treatment of Tazocin (4.5 g q8H) and SoluMedrol (40 mg, Bid). This patient was confirmed to have the influenza A virus (subtype H3N2), according to virus culture tests and reverse transcription polymerase chain reaction analyses (RT-PCR) of the patient’s nasopharyngeal secretions, performed in the lab of the state’s center for disease control and prevention (CDC).

On her fourth day taking oseltamivir, the patient’s family noticed that she was suffering from hallucinations and interrupted delirium, and exhibiting unusual behaviors such as removing her own infusion pipe. She insisted that somebody was calling her to go to heaven, and that all of the doctors and nurses were malicious. She also presented with shakiness of both her upper extremities and her teeth from time to time. This lasted for seconds at a time and she was able to control the shakiness consciously, without foam at the mouth. She did not suffer from incontinence nor convulsions. She developed further symptoms including delusion and impaired calculation ability, however showed normal response intermittently, especially during the administration of benzodiazepine infusion (diazepam). The neuropsychiatric symptoms did not fluctuate nor deteriorate according to oseltamivir dosing times, and the patient continued to receive oral oseltamivir to fight the influenza A virus.

Four days after initiating treatment, the patient’s fever subsided and the respiratory symptoms alleviated. The oral oseltamivir was discontinued at that point. Her neuropsychiatric symptoms began to improve within a few days, and she was able to give correct answers to questions about her name, her families’ names, her age, and her location, as well as to perform simple calculations (additions and subtractions). She recovered completely within 10 days of the cessation of oseltamivir, and with continued administration of benzodiazepine for 5 days during treatment.

## Discussion and conclusions

Oseltamivir is a neuraminidase inhibitor, which prevents the release of the infectious influenza virus particles from the infected respiratory tract cells of the patients. It can relieve the symptoms, shorten the disease course, reduce the complications and transmission of influenza [5, 6]. Oseltamivir has now been approved for treatment of influenza in adults as well as children by U.S. food and drug administration (FDA), and it is regarded as the most efficacious oral anti-influenza drug by far [7, 8].

Generally speaking, olsetamivir is safe and well tolerated in children and adults with a low risk of side effects, confirmed by the plethora of evidence and clinical studies [9, 10]. The most common side effects presented are fatigue, headache and gastrointestinal reactions including nausea and vomiting, reported by 448 of 1033 patients in a meta-analysis, which usually start in the early period of treatment and resolve spontaneously in a short time [11]. Although rare, in recent years, there have been several case reports of neuropsychiatric events occurring during or after oseltamivir treatment, in Japan and other countries [3, 4, 10, 12, 13]. The most reported neuropsychiatric events are behavioral disorders and delusions/perceptual disturbances, while delirium or delirium-like events are also very common, as reported in a review by Toovey et al. [13]. The unusual behaviors exhibited by patients in these case reports include delirious speech, abrupt anger, frightening episodes, and putting unusual objects into the mouth [14]. Cases from Japan consist of neuropsychiatric events like jumping or falling from a height. Most patients reported presented with no premorbid medical or psychiatric history. Psychiatric side effects were more common in infants and children aged 16 years or younger than in adults (1808 children vs. 658 adults), as reported in a comprehensive review [13]. Therefore, several relevant administrative authorities, including the U.S. Food and Drug Administration (FDA) and the Japanese Ministry of Health, Labour and Welfare (MHLW), have warned against the potential risk of neuropsychiatric adverse events (NPAEs) following oseltamivir use.

In the present case, we describe a 57-year-old female patient who complained of abnormal behaviors, delirium-like events and hallucination from the 4th day after oseltamivir treatment began. Neither did the patient nor her family members had premorbid a psychiatric history, including any similar symptoms presented in this case or any other psychiatric events assessed by psychiatric interviews. The examination results of cerebrospinal fluid (CSF) of this patient, including the opening pressure, the color, cell count, specific gravity, chloride, glucose, proteins, immunoglobulins, were all normal. The antibodies and virus detection via PCR of herpes simplex virus (HSV) type I and II, cytomegalovirus and epstein-barr (EB) virus were all negative. The culture of CSF for bacteria and fungus was also negative. The patient received Tazocin and SoluMedrol in the course of disease, and there have been psychiatric adverse events reported in the use of glucocorticoids and antibiotic agents indeed. However, no apparent correlation between these two drugs and the psychiatric events was observed in this case regarding treatment times. When the treatment of oseltamivir ended and all the other medication continued, the psychiatric symptoms of the patient got better day-by-day and ultimately recovered completely. Although it is difficult to ascertain, taking all these factors into account, we postulate that the neuropsychiatric symptoms of this patient were related to oseltamivir.

Nevertheless, there is still controversy around whether there is correlation between oseltamivir administration and psychiatric adverse events. First, a plethora of evidence shows that Tamiflu is safe and many studies including large numbers of patients have reported no adverse psychiatric events. A meta-analysis for many clinical trials comparing oseltamivir with placebo for treatment of seasonal influenza in adults, including 4328 patients, reported no adverse reactions such as neurological or psychiatric disorders or serious adverse events [9]. However, it is possible that such side-effects could occur rarely in individuals, including mature adults, and/or cases that were missed or not revealed by those previous studies. Second, a number of epidemiological studies, controlled studies, and commentaries indicate that there is no relationship between oseltamivir use and psychiatric adverse events [13, 15, 16]. Third, patients with influenza are in cline to suffer from encephalitis or encephalopathy, symptoms of which are difficult to distinguish from that of oseltamivir-related psychiatric adverse effects [17]. Compared to those receiving placebo or no antiviral drugs, patients receiving oseltamivir showed no higher incidence of neuropsychiatric events, according to prospective clinical studies [13, 18]. These studies suggest that influenza itself, instead of oseltamivir, is more likely to lead to such neuropsychiatric events. Fourth, it’s hard to exclude the confounding factors as a large number of patients received various medications simultaneously, or some other underlying cause for the psychiatric behavior other than the flu drug tamiflu might have been present. There are also other concerning research on this issue. One study suggests that when used for preventative measures, oseltamivir increases the risk of psychiatric events [19]. Most reports of neuropsychiatric events with oseltamivir in children and adolescents are from Japan, which has the highest usage of oseltamivir worldwide [20]. Meanwhile, a research found that the concentration of oseltamivir in the blood of 8-week-old rats was considerably higher than in the blood of 2-week-old rats [21]. Accordingly they suggested that if such developmental changes of prodrug/drug concentration ratio occur in humans, it might provide a rational basis for the putative central nervous system (CNS) side effects observed in young patients. However, some reports have also found that oseltamivir is not associated with an increased risk of psychiatric events in teenagers [13, 18]. Further investigation is required to resolve this debatable issue.

To our knowledge, this is the first reported case of oseltamivir-associated neuropsychiatric events occurring in a mature patient over 50 years old. Given that a seasonal influenza outbreak generally occurs each year, the administration of oseltamivir has become more widespread. The trade-off between benefits and harms should be borne in mind when making decisions to use oseltamivir. Generally speaking oseltamivir is safe and well-tolerated in treatment of influenza and there have been no conclusive studies on the neuropsychiatric side effects of oseltamivir. However, we hope that our case report will lead clinicians to be mindful about oseltamivir’s potential neuropsychiatric side effects, and to pay special attention to each patient’s mental state, both in children and adults.
